# Mental Health in Children with Cerebral Palsy: Does Screening Capture the Complexity?

**DOI:** 10.1155/2013/468402

**Published:** 2013-04-03

**Authors:** H. M. Bjorgaas, I. Elgen, T. Boe, M. Hysing

**Affiliations:** ^1^Department of Paediatric Habilitation, Stavanger University Hospital, Postboks 8100, 4068 Stavanger, Norway; ^2^Department of Clinical Medicine, University of Bergen, Postboks 7800, 5020 Bergen, Norway; ^3^Department of Child and Adolescent Psychiatry, Haukeland University Hospital, Jonas Liesvei 65, 5021 Bergen, Norway; ^4^Regional Centre for Child and Youth Mental Health and Child Welfare, Uni Health, Uni Research, Postboks 7810, 5020 Bergen, Norway; ^5^Institute of Biological and Medical Psychology, Faculty of Psychology, University of Bergen, Jonas Liesvei 91, 5009 Bergen, Norway

## Abstract

*Introduction*. Children with cerebral palsy (CP), one of the most common childhood neurological disorders, often have associated medical and psychological symptoms. This study assesses mental health problems compared to population controls and the ability of a mental health screening tool to predict psychiatric disorders and to capture the complexity of coexisting symptoms. *Methods*. Children with CP (*N* = 47) were assessed according to DSM-IV criteria using a psychiatric diagnostic instrument (Kiddie-SADS) and a mental health screening questionnaire (SDQ). Participants from the Bergen Child Study, a large epidemiological study, served as controls. *Results*. Children with CP had significantly higher means on all problem scores including impact scores. Two in three children scored above 90th percentile cutoff on Total Difficulties Score (TDS), and 57% met criteria for a psychiatric disorder, yielding a sensitivity of 0.85 and a specificity of 0.55. Mental health problems coexisted across symptom scales, and peer problems were highly prevalent in all groups of psychiatric disorders. *Conclusion*. A high prevalence of mental health problems and cooccurrence of symptoms were found in children with CP compared to controls. Screening with SDQ detects mental health problems, but does not predict specific disorders in children with CP. ADHD is common, but difficult to diagnose due to complexity of symptoms. Mental health services integrated in regular followup of children with CP are recommended due to high prevalence and considerable overlap of mental health symptoms.

## 1. Introduction

### 1.1. Cerebral Palsy (CP)

CP is one of the most common neurodevelopmental conditions in childhood, affecting 2-3/1000 [1, 2]. While motor impairment is the diagnostic basis, the disorder often presents with associated symptoms and a wide range of related impairments such as epilepsy, pain, and cognitive and communicative impairments. Mental health problems as another main associated symptom, have recently gained awareness, as assessed by screening questionnaires [3–6] and by diagnostic interviews [7]. Using a diagnostic interview, one in two children with CP met criteria for a psychiatric disorder, of which attention deficit hyperactivity disorder (ADHD/ADD) was the most common. Additionally, one in five children had more than one diagnosis, and the presence of psychiatric disorders was not significantly determined by type and severity of the CP condition [7]. The complex symptom presentation is in accordance with the Early Symptomatic Syndromes Eliciting Neurodevelopmental Clinical Examination (ESSENCE) model that emphasizes the coexistence of symptoms in multiple domains in neurodevelopmental disorders [8]. Further, there is growing evidence that many children with neurodevelopmental disorders are more prone to psychiatric disorders in adulthood, some of which can be screened for and treated in childhood [9, 10]. While there is growing awareness about the complexity of the CP condition, most of the health services are provided in specialized paediatric clinics with motor impairments as the main focus. To detect mental health problems in children with CP, screening questionnaires may be useful. However, little is known regarding the ability of these questionnaires to disentangle the complexity of mental health problems in children with CP.

### 1.2. Mental Health Screening

Questionnaires such as the Child Behaviour Checklist (CBCL) [11] and the Strengths and Difficulties Questionnaire (SDQ) [12, 13] have both been used to describe the prevalence of mental health symptoms in children with CP [3, 4]. An Icelandic study using CBCL and comparing 36 preschool children having CP with a randomly selected sample of preschool children found that 40–50% of children with CP suffered substantially from behavioural and emotional problems as reported by parents, demonstrating the early coexistence of these symptoms as suggested by the ESSENCE model [4, 8]. A high prevalence of diverse mental health problems have also been found in other studies including school age children when using the SDQ [3, 14, 15]. Children with CP scoring above abnormal range were found in one of four children in a European multicentre study, and in a recent Canadian study [3, 15], and an even higher number in a study including children with CP hemiplegia only, a subtype generally less severely affected [14]. All three studies has used British comparison norms. Peer problems were found to affect more than one third of children with CP in these studies. Likewise, hyperactivity problems were found in one of four children, conduct problems in one of six children, and emotional problems ranging from 17% to 32%. These findings indicate a high prevalence of mental health problems in children with CP and a considerable coexistence of problems. 

### 1.3. The Strengths and Difficulties Questionnaire

The SDQ is a short 25-item screening tool to detect mental health problems and prosocial behaviour in children. In combination with the impact scores, which depict the impairment from mental health problems in response to family, social, and school situations, it has shown accuracy in detecting psychiatric disorders, and has been thoroughly validated both in general populations, and in populations of children suffering from chronic illnesses [12, 13]. Psychometric properties of the Total Difficulties Score (TSD) of the SDQ compared to the Development and Well-being Assessment (DAWBA) has shown a sensitivity level of 0.63 in the general British childhood population, up to 0.87 in at-risk populations, likewise specificity has shown values from 0.47 in an at-risk populations, to 0.95 in a general British childhood population [12, 16, 17]. To our knowledge, none of the previously used questionnaires have yet been validated against a diagnostic interview in a population of young children with CP, despite knowledge that there is a considerable overlap of mental health symptoms in addition to a complexity of somatic symptoms which may affect the psychometric properties of the screening tool.

### 1.4. Using the ESSENCE Model as a Framework

We wanted to assess mental health problems in children with CP compared to population-based controls and to assess frequency and coexistence of symptoms. Secondly, we wanted to assess the ability of a mental health screening instrument (SDQ) to sufficiently detect prevalence and coexistence of mental health problems in children with CP, comparing SDQ findings to results from a diagnostic psychiatric interview (the Kiddie-SADS).

## 2. Methods

### 2.1. Population

A cohort of children with cerebral palsy (CP) in the three western counties of Norway born 2001–2003 were invited to participate in the study. Cerebral palsy (CP) was stated for 101 children prior to the study. Three of the children had however subsequently been rediagnosed and given a different neurological diagnosis and were therefore excluded from the study. Of the 98 children invited, 67 (68%) participated and were examined regarding psychiatric disorder. For 11 children however, diagnosing a psychiatric disorder using a diagnostic interview was inappropriate due to severe disability, and these children were omitted from the study. The remaining 56 children were included in the present study. The population has been described in detail in an earlier study [7].

The Bergen Child Study (BCS) served as control group [18]. This study is a large longitudinal population-based study involving all children (9155) in the two Norwegian municipalities Bergen and Sund with matching parent SDQ obtained from 6297 children. It has been described in detail elsewhere [19], and we will only give a brief description here. The whole population was assessed for mental health problems using the Strengths and Difficulties Questionnaire (SDQ) as well as answering a question about chronic illness or disability. Data collected when the children were 7–9 years old were used as comparisons for the present study.

### 2.2. Classification, Functional Levels, and Medical Information

Cerebral palsy was classified according to ICD-10 criteria with the following subgroups: spastic bilateral and unilateral, dyskinetic, atactic, or not further classified. Functional level was classified by the Gross Motor Function Classification System (GMFCS) and Manual Ability Classification System (MACS) which distinguishes five levels (I–V), with level V being the most severe [20, 21]. The cognitive level was recorded through information in the medical record, or through the educational system, and was verified by parents during the interview if results from updated cognitive tests were not available. The population is described in detail in a previous study [7]. 

### 2.3. Mental Health: SDQ

The Strengths and Difficulties Questionnaire (SDQ) consists of 25 items, of which four record problem domains, each including five items, and one prosocial domain (scale) including five items. Each item can be answered with “not true,” “somewhat true,” or “certainly true” rated 0–2 for negatively worded items, and inversely 2–0 for positively worded items. The problem domains are *hyperactivity problems* including items such as inattentiveness and distractibility, *conduct problems* including items such as disobedience and temper tantrums, *emotional problems* including items such as anxiety and worry, *peer problems* including items such as loneliness and preferring adult company. *Prosocial behaviour* consists of items such as being helpful and kind. 

Combining the four problem subscales (0–10) computes the Total Difficulties Score (TDS) (0–40). The SDQ also includes an impact score (IS) which measures the impact of mental health problems. For each of the subscales, a score at or above the 90th percentile of the controls was defined as screen positive and a Total Difficulties Score at or above the 90th percentile as risk of having a psychiatric disorder.

### 2.4. Psychiatric Disorders: Kiddie-SADS

Parents of children with CP were interviewed using the Schedule for Affective Disorders and Schizophrenia for School-Age Children: Present and Lifetime Version (6-18) 10.04.00 (Kiddie-SADS), a semistructured child psychiatric diagnostic interview designed to unveil psychiatric symptoms within the following groups of disorders: affective, anxiety, psychotic, eating, attention/hyperactivity, oppositional defiant, conduct, tics, substance abuse, and posttraumatic stress disorders, as well as encopresis and enuresis. Diagnostic conclusions were drawn according to the Diagnostic and Statistical Manual of Mental Disorders, Fourth Edition (DSM-IV). Parents were interviewed, and we recorded present symptoms. All interviews were conducted by the first author, a child and adolescent psychiatrist. A *psychiatric disorder* was ascertained if criteria listed in the DSM-IV for each specific diagnosis were fulfilled, including severity and duration of specific symptoms.

### 2.5. Mental Health Screening and Psychiatric Disorder

Mental health problems recorded using the SDQ were compared to psychiatric disorders (DSM-IV criteria) for the following symptom-disorder pairs: SDQ-emotional problems compared to emotional disorders, SDQ-hyperactivity problems compared to ADHD/ADD, and SDQ-conduct problems were compared to ODD and conduct disorders. Finally, the SDQ-Total Difficulties, SDQ-peer problems, and SDQ-impact scores were compared to any psychiatric disorder.

### 2.6. Statistical Analyses

Independent *t*-tests were used to compare mean scores from children in the CP group to those from the general population. *Sensitivity *was defined as the proportion of actual positives which were identified as such. *Specificity* was defined as the proportion of actual negatives which were correctly identified as such. *Positive predictive value (PPV)* was defined as the proportion of positive tests that were true positives, and *negative predictive value* was defined as the proportion of negative tests that were true negatives. *Sensitivity, specificity, PPV, and NPV above 80% were regarded as high. Cross-tabulations and* 90th percentile cutoff were used to calculate these parameters.

Effect size was defined using Cohen's *d* measuring the standardized mean difference between the study population and controls; a large effect size was defined as ≥0.8, a medium effect size as ≥0.5, and a small effect size as ≥0.3. Cross-tabulations were done to evaluate possibly significance between high scorers on SDQ-TDS and types and severity of CP.

A linear regression analysis was used to explore the influence of each of the five symptom items in the peer problem score, the latter being the dependent variable, and the five items were independent variables.

## 3. Results

### 3.1. Population

Of the 56 children in the present study, 47 completed the SDQ. The mean age was 7 years and 3 months (87.6 months, SD 6.5). Thirty (64%) were boys, more than half had CP diplegia, and four of five children had Gross Motor Function Classification System (GMFCS) levels I and II. One of five had an intellectual disability, and approximately the same number suffered from epilepsy (Table 1). Type and severity of CP conditions were not significantly associated with high scorers on SDQ-Total Difficulties Score.

### 3.2. Mental Health Problems in Children with Cerebral Palsy Compared to a Population-Based Control

Children with CP had significantly higher mean scores compared to controls, affecting all problem scales as well as impact score (Table 2). A large effect size was found for the Total Difficulties Score, hyperactivity problems, conduct problems, peer problems and impact score, and moderate effect size for emotional problems, and a small effect size for prosocial behaviour (Table 2). 

### 3.3. Coexisting Symptoms

When comparing all SDQ screen positives across the problem scales, including TDS and impact score, with the following four groups of psychiatric disorders, emotional disorders, conduct disorders, ADHD/ADD, and any psychiatric disorders, we found a high prevalence of coexisting screen positives across groups of psychiatric disorders in children with CP (Table 3). There was a discrepancy between screen positives and children meeting criteria for a psychiatric disorder, most prevalent for SDQ-hyperactivity problems (3/24) and SDQ-conduct problems (2/4). For the 24 children meeting criteria for ADHD/ADD, 23 were screen positives for peer problems. To gain understanding of the large proportion experiencing peer problems in the present study, a regression analysis was done to identify the impact of each of the five items in the peer problem scale. We found preference for adult company, the most weighted item, followed by lack of close friendships, loneliness, being bullied, and not being liked in diminishing order.

### 3.4. SDQ and Psychiatric Disorder

Screening efficiency of the SDQ-TDS in children with cerebral palsy was assessed by comparing SDQ screen positives (i.e., scores above 90th percentile) with children meeting criteria for a psychiatric disorder according to Kiddie-SADS (Table 4). Sensitivity for all symptom groups varied between 0.13 and 1.0, and specificity varied from 0.55 to 0.87. For TDS, sensitivity was 0.85 and specificity 0.55 (Table 4). 

## 4. Discussion

### 4.1. Main Findings

Children with CP in the present population based study had significantly higher means on all SDQ subscales compared to the general childhood population. There was considerable mental health symptom overlap across all categories, and peer problems were highly prevalent in all four groups of psychiatric disorders. Sensitivity for TDS was adequate for SDQ compared to any psychiatric disorders using a semistructured child psychiatric diagnostic interview (Kiddie-SADS).

### 4.2. Mental Health Problems in Children with Cerebral Palsy

Compared to controls, mean scores in children with cerebral palsy were significantly higher on all scales, which is consistent with previous findings. Previous studies have found high TDS mean scores 11.0–12.4, which are similar to those found in the present study [3, 6, 14, 15], even when children with GMFCS level V and intellectual disability (ID) were excluded and therefore as expected had less medical complications than a population consisting of all five GMFCS levels [1, 7]. 

Mean scores for emotional problems in the present study were similar to the study by Parkes et al. since 2008 [3] and Brossard-Racine et al. where all CP subgroups were included [15]. In the study by Parkes et al. including children with CP hemiplegia only however [14], a fourfold higher mean score for emotional problems was found. This may indicate that anxiety and depressive disorders are more prevalent in children having CP with a functional level closer to their healthy peers, as they perhaps more often are met with the same expectations in all areas as children without CP, leading to a sense of shortcoming.

For the conduct and hyperactivity subscales, mean scores in the present study were similar (2.5 and 4.3, resp.) to those found in comparable studies, 1.7–2.0 for conduct problems and 4.3–4.8 for hyperactivity problems [3, 14, 15]. This finding underlines a consistently high prevalence of behavioural problems in children with CP, which was also confirmed in a recent meta-analysis where one of four children with CP were found to have behaviour disorder [22]. Mean scores for peer problems however were higher in the present study compared to previous studies of children with cerebral palsy (2.7–3.0) [3, 14, 15]. Peer problems were found in nine of ten children with CP in the present study, whereas Parkes et al. found peer problems in one of three children in their studies and two of five children in the Canadian study by Brossard-Racine et al. Peer problems were highly prevalent across all diagnostic groups, that is, emotional disorders, conduct disorders/ODD, as well as hyperactivity disorders, and could be related symptomatically to autism spectrum disorders (ASD) which have been found to be prevalent in children with CP [23]. Similarly, ASD is often part of neurodevelopmental conditions in general with a considerable overlap of conditions as described in the ESSENCE model [8]. When analysing the peer problems construct in detail, preference for adult company and loneliness were highly prevalent in the present study. This finding is coherent with information given by parents during the Kiddie-SADS interview, where many parents pointed to their children taking part in organized school and leisure activities, although they often had few mutual own-aged friendships. This could indicate that some might be passively watching rather than being active participants in peer activities. Another possible hypothesis for peer problems is slow cognitive processing and speech, which for many children with CP with normal IQ may result in difficulties matching their peers in spontaneous play activities, perhaps resulting in a preference for adults to accompany during activities [24]. 

### 4.3. Behaviour Problems

For ADHD/ADD which was the most prevalent psychiatric disorder in the present study, the screening efficacy using SDQ in the present study was 13%, about half of that found in the studies by Parkes et al. and Brossard-Racine et al. [3, 14, 15], despite a high prevalence of ADHD/ADD (52%) when parents of the same children were interviewed with the Kiddie-SADS and diagnosed according to DSM-IV criteria. On the contrary, symptoms of conduct disorders in the present study were almost twice as high as those found in the studies by Parkes et al. and Brossard-Racine et al. [3, 14, 15], despite a much lower prevalence (8.5%) when diagnosing according to Kiddie-SADS. Other studies have pointed to cooccurrence of symptoms between ODD (oppositional defiant disorders) and ADHD/ADD, emotional problems and peer problems [25]. Although the studies by Parkes et al. and Brossard-Racine et al. include children with all CP subtypes, similar to the present one, mental health symptoms may be recognized as conduct or peer problems rather than hyperactivity problems in the study population. Attention problems may not be recognized as such, as these children in Norway to a large degree have individual support during most of their time at school, and parents are often primed to assist their children with all practical issues at home and may not recognize their children's challenges in memory or attention span. During the semistructured Kiddie-SADS interview, these issues were elaborated, and many children met criteria for attention problems when parents were asked to judge their child's attention span in a situation where they were left to do their school work or organize their belongings as would their peers or siblings. Perhaps the screening tools are more sensitive to symptoms such as peer and conduct problems in a population of children with CP than to hyperactivity problems and therefore may not fully capture the attention and/or hyperactivity problems these children suffer, as motor symptoms due to the CP condition may disguise restlessness and distractibility which may be interpreted differently. 

### 4.4. Coexisting Conditions

In accordance with the ESSENCE model, we found an extensive overlap of mental health symptoms in children with CP meeting criteria for a psychiatric disorder. Gillberg et al. found a high prevalence of other psychiatric disorders such as emotional problems and ODD in children with ADHD [26], which has also been found in a previous study including children in the present study [7]. Similarly, ADHD was found in half of all children with mild mental retardation MMR in association with other developmental problems [27]. ADHD seems to be a common associated factor in many different developmental disorders or neurological conditions stemming from the brain and may be associated with the immature nervous system being vulnerable to brain injury in the neonatal period. Perhaps this is one of the reasons for a high level of diversity and overlap of mental health symptoms in children with disorders such as CP which occurs in the neonatal period and why mental health symptoms in children with CP seem to present differently from the general childhood population. It may also explain why the SDQ seems to be a less accurate screening tool for ADHD/ADD in children with CP than one would expect from studies conducted in a general childhood population. The extensive overlap of symptoms constitutes a considerable challenge both in providing diagnostic tools and in gaining competence in recognizing psychiatric disorders in children with a complexity of interacting symptoms.

### 4.5. Screening Efficiency of the SDQ

The child psychiatric diagnostic interview Kiddie-SADS and the Strengths and Difficulties Questionnaire were both used to assess the same children which enabled us to evaluate the psychometric properties of the SDQ when using the Kiddie-SADS as the gold standard. When comparing specific mental health symptoms and psychiatric disorders, we found a large range in sensitivity and specificity between the least and the most sensitive symptom-disorder pairs in this study. Sensitivity for TDS compared to any psychiatric disorder according to Kiddie-SADS in the present study was 0.85 and specificity 0.55, which differ from those found in the general childhood population yielding sensitivity of 0.63 and specificity of 0.95 [12]. For a population of children referred to mental health services in Norway, sensitivity was 0.85 and specificity 0.52 for TDS [16], and for children with chronic illnesses in a Norwegian study, sensitivity was 0.81 and specificity 0.69 for TDS [17] which is comparable to the present study. For emotional, conduct, and hyperactivity disorders, however, we found that the positive predictive value was low, indicating that there may be a need for caution when using the SDQ to predict these disorders in children with CP in early childhood.

### 4.6. Clinical Implications

In the present study, 57% met criteria for a psychiatric disorder, and 67% were screen positive on Total Difficulties Score (TDS). Sensitivity of the SDQ for TDS may be considered adequate however predictive value for specific psychiatric disorders is found to be inadequate in the present study. Knowing that mental health problems seem to affect two in three children with CP, with a considerable overlap of mental health symptoms and psychiatric disorders, we are faced with challenges in disentangling and diagnosing a conglomerate of symptoms. Establishing mental health services as part of the regular follow-up program for children with CP seems relevant. In addition, using the SDQ as a screening instrument to identify children with CP without mental health problems, not in need for mental health services, may be legitimate, bearing in mind, however, the high rate of psychiatric diagnosis and the low diagnostic specificity especially for AD/HD which is one of the most frequent diagnosis. 

### 4.7. Limitations

The version of Kiddie-SADS used in the present study did not contain a section on autism spectrum Disorders (ASD), which is a weakness, since all children diagnosed with a psychiatric disorder were screen positive for peer problems. Further, one of three had peer problems without having a psychiatric disorder, and these might represent children with an ASD. Likewise, we did not use the SDQ algorithm for predicting psychiatric disorders as we had a single informant.

A small study population was also a limitation to the study. Similarly, omitting children with GMFCS V and ID from the study may represent a weakness as information on mental health among severely affected children is lacking. Previous studies have attempted to include the most severely affected children; however, it has not been possible to draw diagnostic conclusions regarding psychiatric disorders due to the complexity of motor, cognitive, somatic, and epileptic disorders affecting these children [7, 28]. The current study was however representative of children with CP with GMFCS levels I–IV. 

### 4.8. Conclusions

The present study supports the previous literature indicating a high prevalence of mental health problems in children with CP compared to controls. The SDQ disclosed problems relating to peers in more than three in four children, and further studies regarding ASD are recommended. We recommend establishing mental health services for children with CP as part of the regular follow-up program; however screening children with CP for mental health problems in the paediatric clinics using the SDQ seems relevant when established mental health service programs are not available.

## Figures and Tables

**Table 1:** Demographics and functional data for a population of children with cerebral palsy living in the Western Health Region of Norway at school starting age.

	*N* (%)
*N* = 47	
Boys	30 (64)
Cerebral palsy subtype	
Bilateral	25 (53)
Unilateral	18 (38)
Ataxia/dyskinesia	4 (9)
GMFCS level^i^	
I-II	38 (81)
III-IV	9 (19)
Intellectual disability^ii^	10 (21)
Epilepsy	9 (19)
Visual impairment^iii^	19 (40)

^i^Gross Motor Function Classification System.

^ii^Defined as IQ < 70.

^iii^Defined as any visual impairment.

**Table 2:** Mental health for children with cerebral palsy (CP) using mean scores of the Strengths and Difficulties Questionnaire compared with controls.

	CP group (47)		Controls^a^ (7007)				95%	CI	Cohen's *d*
	Mean	SD	Mean	SD					
Emotional problems	2.6	2.2	1.3	1.7	4.0 (46)	<.001	0.6	1.9	0.66
Conduct problems	2.5	1.5	1.0	1.3	6.7 (45)	<.001	1.1	2.0	1.07
Hyperactivity problems	4.3	1.5	2.7	2.1	7.8 (47)	<.001	1.2	2.1	0.88
Peer problems	4.5	1.5	1.0	1.5	16.0 (46)	<.001	3.1	4.0	2.33
Total difficulties scores	13.9	5.3	5.6	5.0	10.7 (46)	<.001	6.8	9.9	1.61
Prosocial behaviour^b^	7.8	2.1	8.5	1.5	−2.2 (45)	0.03	1.3	−0.1	0.39
Impact score	2.9	3.8	0.4	1.5	4.6 (46)	<.001	1.4	3.7	0.94

^a^Bergen Child Study,^ b^higher scores indicate more prosocial behaviour.

**Table 3:** Coexisting mental health symptoms in children with cerebral palsy meeting criteria for a psychiatric disorder according to DSM-IV criteria assessed by Kiddie-SADS.

	Children scoring above 90th percentile cutoff on the Strengths and Difficulties Questionnaire					
	Emotional problems	Conduct problems	Hyperactivity problems	Peer problems	TDS^a^	Impact score
Psychiatric disorder						
Emotional (*N* = 5)	5/5	3/5	1/5	5/5	5/5	3/5
Conduct/ODD^b^ (*N* = 4)	2/4	2/4	2/4	4/4	4/4	4/4
ADHD/ADD^c^ (*N* = 24)	9/24	10/24	3/24	23/24	19/24	19/24
Any psych^d^ (*N* = 27)	12/27	12/26	4/27	26/26	22/26	20/27

^a^TDS: Total Difficulties Score; ^b^ODD: oppositional/defiant disorder; ^c^ADHD: attention-deficit/hyperactivity disorder; ADD: attention deficit disorder; ^d^Any psych: any psychiatric disorder.

**Table 4:** Mental health screening compared to having a psychiatric disorder (according to DSM-IV criteria assessed by Kiddie-SADS) for children with cerebral palsy at school starting age.

SDQ-symptoms versus psychiatric disorders	Above 90th percentile on SDQ^a^ *N *(%)	Psychiatric disorder present *N* (%)	Sensitivity	Specificity	PPV	NPV
Emotional symptoms versus emotional disorders^b^	14 (29.8)	5 (10.6)	1.00	0.79	0.36	1.00
Conduct problems versus conduct disorder/ODD^c^	16 (34.8)	4 (8.5)	0.50	0.67	0.13	0.93
Hyperactivity problems versus ADHD/ADD^b^	6 (12.8)	24 (51.1)	0.13	0.87	0.50	0.49
Total Difficulties Score versus any psychiatric disorder^c^	31 (67.4)	26 (56.5)	0.85	0.55	0.71	0.73
Peer problems versus any psychiatric disorder	41 (89.1)	26 (56.5)	1.0	0.25	0.63	1.0
Impact score versus any psychiatric disorder	27 (57.4)	27 (57.4)	0.74	0.65	0.74	0.65

PPV: positive predictive value; NPV: negative predictive value; ^a^SDQ: strengths and difficulties questionnaire ^b^ *N* = 47; ^c^ *N* = 46.
